# Dual-Functional Utilization of Phosphogypsum as Cementitious Binder and Aggregate in Concrete: Interfacial Compatibility and Feasibility Analysis

**DOI:** 10.3390/ma19020398

**Published:** 2026-01-19

**Authors:** Pan Chen, Zhexin Wang, Feng Zhu, Shujie Wan, Mengyang Huang, Pengfei Liu, Dongxu Zhang, Cai Wu, Yani Lu

**Affiliations:** 1 School of Civil Engineering, Hubei Engineering University, Xiaogan 432000, China; chenpan@hbeu.edu.cn (P.C.); zhufeng225@163.com (F.Z.); wanshujie0911@163.com (S.W.); huangmengyang1025@163.com (M.H.); liupengfei0821@163.com (P.L.);; 2 Hubei Province Engineering Research Center for Cement-Based Ultra-High Performance Concrete and Prefabricated Building Technology, Xiaogan 432000, China

**Keywords:** phosphogypsum concrete, cementitious binder, phosphogypsum aggregates, mechanical properties, microstructure

## Abstract

Addressing the environmental challenges posed by phosphogypsum (PG) stockpiling, this study investigates the synergistic mechanisms of a dual-functional application strategy where PG serves as both cementitious binder and aggregate. Unlike previous research limited to single-mode utilization, this study focuses on the interfacial compatibility between PG-based binders and PG aggregates (PGA). Through a comparative experimental program, the mechanical performance and microstructure of different binder–aggregate combinations were evaluated. The proposed dual-functional formulation achieved a high PG incorporation rate of 38% by mass. While the compressive strength of 39.3 MPa was lower than that of the reference ordinary concrete, it comfortably surpasses the C30 strength requirement for structural applications, validating its engineering feasibility. Comparative analysis revealed that although natural stone aggregates possess higher intrinsic strength, the PG-binder/PGA system exhibits superior interfacial bonding compared to the PG-binder/stone system. Microstructural observations indicated that this synergistic interaction facilitates the formation of interwoven ettringite and C-S-H gel networks, contributing to a structurally integrated interfacial transition zone (ITZ). These findings suggest that the dual-functional strategy offers a viable pathway for developing low-carbon building materials by balancing high-volume waste utilization with mechanical compliance.

## 1. Introduction

Phosphogypsum (PG), the primary solid waste from the phosphorus chemical industry, has a global annual production exceeding 300 million tons. The open-air stockpiling of PG creates severe environmental hazards, including heavy metal leaching and land occupation, representing a critical global challenge [1,2,3,4]. Simultaneously, the concrete industry is under immense pressure to reduce its reliance on natural resources and lower carbon emissions. Consequently, creating a closed-loop system where industrial waste substitutes both the binder and the aggregate in concrete has emerged as a paramount pathway for sustainable development [5,6,7]. However, realizing this dual-substitution vision is hindered by the complex physicochemical properties of PG, which often lead to conflicts between high waste utilization rates and mechanical reliability [8,9].

The application of PG in cementitious binders has been widely explored as a primary approach to solid waste resource utilization. However, a recurring theme in existing literature is the trade-off between substitution quantity and performance stability. Studies have shown that untreated PG used alone as a cementitious binder suffers from defects such as low early strength and prolonged setting time. Islam et al. [10] reported that when raw PG replaced 10% natural gypsum, the 28-day compressive strength of Portland cement reached 29.5 MPa, but substituting more than 15% caused a 22% strength loss due to residual impurities. While optimization strategies exist, such as those by Hu et al. [11], who successfully prepared C30-grade PG concrete by optimizing the water-cement ratio and silica fume content (achieving 45.1 MPa), material variability remains an issue. Levickaya et al. [12] noted that PG could exhibit greater compressive strength than natural gypsum at low water–solid ratios because of its lower degree of particle agglomeration. Nevertheless, volume stability issues persist. Yang et al. [13] developed nonautoclaved aerated concrete by increasing the sulfate reactivity of PG with calcium sulfoaluminate cement, but the drying shrinkage rate far exceeded standard limits. These studies collectively indicate that while raw PG possesses hydration potential, its standalone application in binders is limited by the need for costly pretreatment or activators to balance mechanical performance.

To overcome the limitations of single-component systems, composite cementitious systems have been proposed to enhance PG utilization efficiency through the synergistic effects of multiple solid wastes. Gong et al. [1] employed ultrasonic cyclic water washing to pretreat PG, where secondary hydration-generated ettringite increased the compressive strength of the composite cementitious binder by 12%. However, when the substitution exceeded 30%, unreacted gypsum crystals caused pore coarsening. Similarly, Pratap [14] reported that microsilica and PG synergistically promoted the formation of C-A-S-H gels, reducing the chloride ion permeability of geopolymer concrete, though the radioactive risks remained unassessed. Kong et al. [15] constructed a ternary system of red sandstone-PG-cement, revealing that Ca^2+^ and SO_4_^2−^ in PG could activate the Al^3+^ reactivity of red sandstone, yet the strength reached only 32.1 MPa, restricting the substitution ratio to ≤25%. High-volume attempts often compromise strength significantly. For instance, Wang et al. [16] developed autoclaved aerated concrete panels with 75% solid waste content but achieved a compressive strength of only 5.2 MPa. Feng et al. [17] introduced alkali modulus regulation in a ternary system of PG–fly ash–slag, increasing the axial compressive strength by 17%, but high substitution led to a 30% increase in porosity. Evidently, although multisource solid waste synergy can improve chemical reactivity, a recurring trade-off persists between high substitution rates and microstructural density.

Further research has focused on chemical modification to mitigate these performance shortcomings. Fu et al. [18] enhanced the freeze–thaw cycle strength retention rate of a PG-based cementitious binder by 40% through the combined addition of quicklime and silica fume, but excessive quicklime caused expansion cracks. Tian et al. [19] used calcium sulfoaluminate cement to reduce the setting time to 102 min, but the drying shrinkage rate still exceeded the standard limit by sevenfold, necessitating optimized curing regimes. Additionally, Huang et al. [20] developed high-volume PG foamed concrete using water glass to activate slag reactivity, which resulted in a strength of only 3.36 MPa. Despite these technological advances, relying solely on cementitious substitution appears to have reached a saturation point in terms of mass utilization. To achieve significantly higher consumption, the focus must expand beyond the binder phase to the volumetric bulk of concrete: the aggregates.

The preparation of PG into construction aggregates offers an alternative pathway to alleviate natural resource shortages and achieve large-scale waste utilization. Ding et al. [21] used cold-bonding technology to prepare PG aggregates (PGA) and reported that when the PG content was ≤80%, the 28-day cylindrical compressive strength of the aggregates reached 11.9~16.5 MPa, with a softening coefficient ≥ 0.82, meeting lightweight aggregate standards. This process generated ettringite and C-S-H gels that encapsulated unreacted particles, significantly reducing the leaching concentrations of phosphorus and heavy metals. However, when the PG content exceeded 80%, the porosity and water absorption of the aggregates sharply increased, leading to mechanical performance degradation. This suggests that while physical encapsulation is effective, the inherent mechanical weakness of porous PG aggregates remains a key hurdle for structural applications.

Therefore, to maximize utilization, PG aggregates must be chemically compatible with the cementitious system. Sun et al. [5] developed a high-volume PG concrete (HPGC) system consuming 800 kg of PG per cubic meter by using PG as both a cold-bonded aggregate and a raw material for supersulfated cement, achieving strengths of 18~40 MPa. Addressing fine aggregates, Sun et al. [22] further investigated PG-based cold-bonded fine aggregates in excess sulfate cement concrete (ESCC), noting that while replacing natural sand reduced strength by 17.2%, mix optimization could still meet the 30 MPa requirement.

Although these studies have demonstrated the feasibility of using PG as both binder and aggregate, the fundamental synergistic mechanism, specifically the interfacial compatibility between the PG-based binder and PG aggregates, remains under-explored. Previous research has largely focused on mix proportion optimization or macroscopic properties [23,24]. Crucially, few studies have systematically investigated the specific chemical affinity within this homologous system compared to the traditional heterogeneous system (PG binder with silicate stone). Conventional stone aggregates often exhibit poor bonding with PG-based binders due to chemical incompatibility, resulting in weak Interfacial Transition Zones (ITZ). It remains unclear whether the chemical homology between a PG binder and a PG aggregate can effectively repair these weak interfaces and compensate for the aggregate’s intrinsic strength defects.

This study proposes that utilizing PGA with a PG-based binder creates a chemically compatible system that can mitigate the severe performance degradation typically seen in high-volume waste concrete. Instead of relying on traditional empirical mix optimization, this research employs a comparative mechanism analysis to decouple the effects of binder and aggregate types, focusing on the ITZ evolution and its role in maintaining sufficient mechanical strength for structural feasibility. The research framework encompasses material characterization, mix proportion design, mechanical testing, and microstructural analysis, establishing a balanced relationship between PG utilization strategies and concrete performance metrics.

## 2. Materials and Methods

### 2.1. Experimental Raw Materials

PG was supplied by Jiuqi Building Materials Co., Ltd., (Zhucheng, China). Prior to use, organic matter and soluble impurities were removed from the PG via water washing, reducing the P_2_O_5_ mass fraction to less than 0.1%. The PG is a grayish-white powder with a specific surface area of approximately 500 m^2^/kg and a ture density of 2.4 g/cm^3^. Its primary physical characteristics are summarized in Table 1.

**Figure 1:**
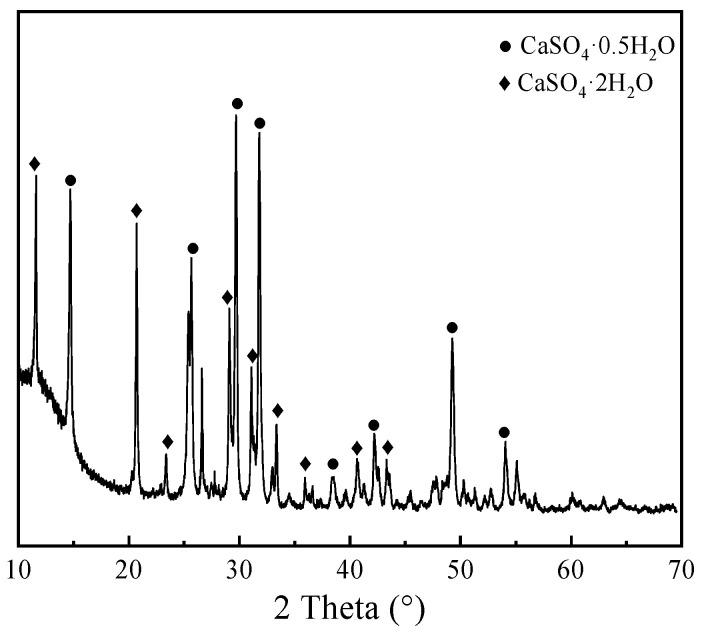
XRD patterns of PG.

X-ray diffraction (XRD) analysis was conducted to investigate the crystalline phase composition of PG, as illustrated in Figure 1. The results identify calcium sulfate hemihydrate (CaSO_4_·0.5H_2_O) and calcium sulfate dihydrate (CaSO_4_·2H_2_O) as the predominant mineral phases. The sharp and well-defined diffraction profile indicates that the PG phases possess a high degree of crystallinity, which is favorable for subsequent hydration reactivity. The specific diffraction angles correspond to standard crystallographic data for gypsum phases, confirming the absence of significant impurity interference.

**Figure 2:**
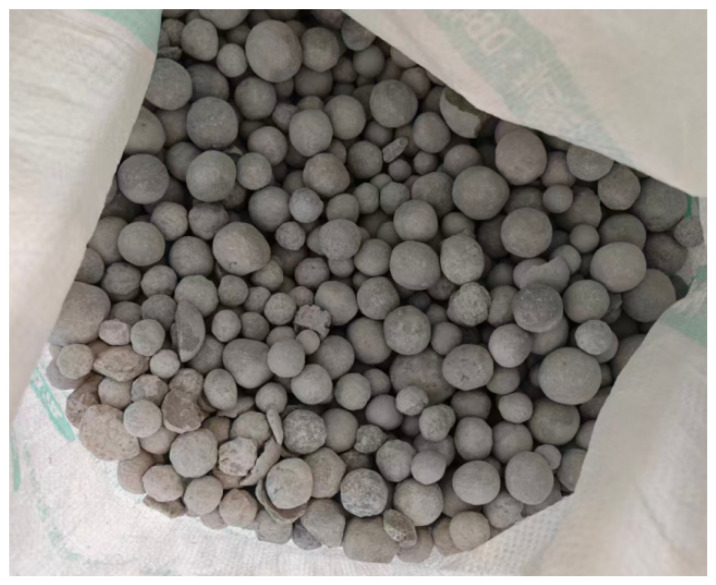
PGA.

The PGA was supplied by Hubei Jvhai Environmental Technology Co., Ltd. (Xiaogan, China), as shown in Figure 2. PGA is produced by high-temperature calcination of raw PG in a rotary kiln to eliminate or neutralize harmful substances and impurities such as phosphorus and fluorine, followed by a disc pelletization process. The calcined phosphogypsum powder was processed in a disc granulator, where atomized water was sprayed to induce partial hydration and agglomeration. This process utilizes the rapid setting characteristics of the hemihydrate phase to form spherical granules without the need for additional binders. These granules were subsequently cured to achieve the final strength. In this study, a PGA with a particle size range of 9–15 mm was employed, which exhibited a grayish-white spherical morphology with a smooth and rounded surface and an apparent density of 1.75 g/cm^3^.

**Figure 3:**
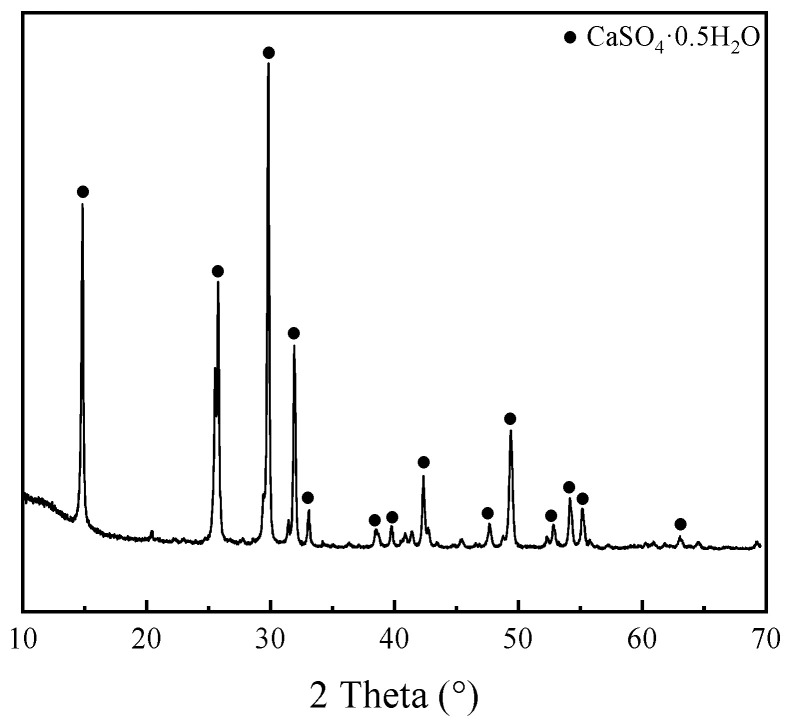
XRD patterns of PGA.

Figure 3 displays the XRD pattern of PGA, revealing that CaSO_4_·0.5H_2_O was the predominant mineral phase. The sharp diffraction peaks observed in the pattern indicate a well-crystallized structure. Although it is derived from the same raw PG material shown in Figure 1, PGA has a more homogeneous crystalline composition and higher purity of calcium sulfate hemihydrate after high-temperature calcination.

**Figure 4:**
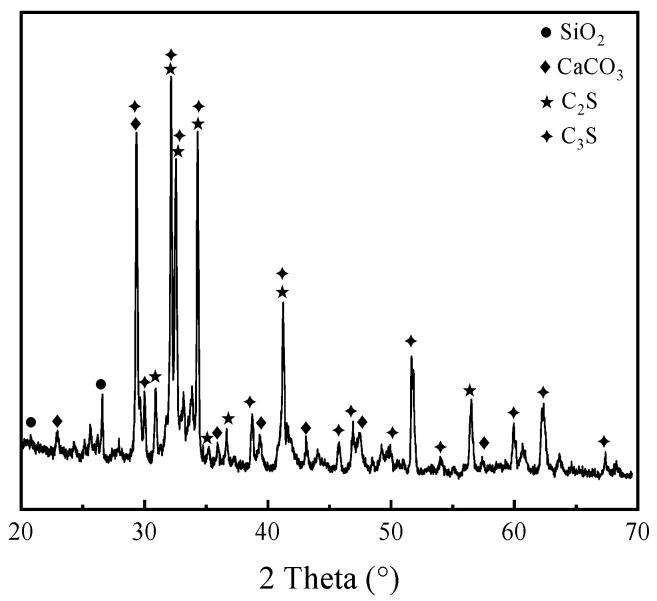
XRD pattern of the cement.

The cement used is 42.5R grade ordinary Portland cement, with a specific surface area of 385 m^2^/kg and a density of 3.15 g/cm^3^. According to the Chinese standard GB 175-2023 [25], the compressive strengths at 3, 7, and 28 days are 18.7, 29.8, and 44.5 MPa, respectively. Figure 4 presents the XRD pattern of the cement used. The mineralogical composition is typical for ordinary Portland cement, dominated by tricalcium silicate (C_3_S) and dicalcium silicate (C_2_S), which govern the early and late-stage strength development, respectively. A minor calcium carbonate (CaCO_3_) phase was also detected. The diffraction peaks correspond well with standard patterns for silicate phases, confirming the cement’s suitability as a stable reference binder and activator for the PG system.

The other raw materials used in this experiment include crushed stone and sand, with the sand having a fineness modulus of 2.38. The admixtures include retarders and superplasticizers. The retarder used was a commercial gypsum retarder supplied by Beijing Kaili Tianwei Tech & Trade Co., Ltd., (Beijing, China). It is a white, powdery hydroxy carboxylate-based polymer specifically designed to regulate the hydration kinetics of the PG-based binder. Mechanistically, this retarder adsorbs onto the nucleation sites of the hemihydrate gypsum and cement particles, inhibiting the rapid crystallization of ettringite and gypsum during the early stages. This control is critical for preventing flash setting caused by the high sulfate concentration in PG, thereby extending the setting time to a workable range. The superplasticizer employed was SPT-180, a high-performance water-reducing agent manufactured by Guangzhou Suplast Co., Ltd., (Guangzhou, China). This admixture is a liquid solution with a specific gravity of 1.16 and a water reduction rate exceeding 50%. Relying on steric hindrance effects, the SPT-180 effectively disperses the fine PG and cement particles, permitting a significant reduction in water content while maintaining workability, which facilitates a denser particle packing conducive to subsequent strength development and durability.

### 2.2. Sample Design

Four comparative experimental groups (labeled S1 to S4) were designed to investigate the influence of PG on the mechanical properties of concrete when used as both cementitious binder and aggregate. The mix proportions of the raw materials used in the experiments are shown in Table 2.

The water-to-binder ratio for all the samples was set to 0.34. S1 is the reference mix for the dual-functional system, where PG serves as both cementitious binder and aggregate. In S1, PG accounts for 50% of the mass of the cementitious binder, and PGA constitutes 30% of the total volume. These specific proportions were determined based on a series of preliminary experimental trials intended to maximize waste utilization without compromising engineering performance. For the binder, the 50% replacement level represents an optimal threshold; preliminary tests indicated that exceeding this ratio resulted in significant hydration retardation and insufficient early-age strength. Regarding the aggregate, the 30% volumetric replacement was selected to balance the mix proportions and ensure the stability of the fresh concrete, particularly for the homologous PG binder system (S1). Pilot trials revealed that higher PGA volumes led to mixture segregation and reduced workability due to the density difference between the aggregate and the paste. Compared with S1, S2 is identical in all aspects except that the PGA is replaced by an equal volume of stone. The two groups (S1 and S2) are compared to study the effects of replacing PGA with stone on the mechanical properties of the concrete. Compared with S1, S3 is identical in all aspects except that the PG is replaced by an equal mass of cement. S1 and S3 are compared to study the effects of replacing PG with cement as the cementitious binder on the mechanical properties of concrete. Compared with S3, S4 is identical in all aspects except that the PGA is replaced by an equal volume of stone. S3 and S4 are compared to study the effects of replacing PGA with stone when cement is used as the cementitious binder on the mechanical properties of concrete.

A total of 18 cubic specimens (100 mm × 100 mm × 100 mm) were prepared for each mix proportion to evaluate mechanical and physical properties. The specimen allocation was as follows: 9 specimens were used for compressive strength testing at curing ages of 3, 7, and 28 days; 6 specimens were employed to determine the water absorption and softening coefficient, while the remaining 3 specimens served as reserves for physical property verification. Additionally, 3 standard-sized specimens (150 mm × 150 mm × 150 mm) were cast specifically for stress–strain analysis at 28 days. All tests were performed in triplicate to ensure statistical reliability. The results are reported as mean values ± standard deviation (SD). To evaluate the statistical significance of the observed differences between the binder–aggregate combinations, a one-way analysis of variance (ANOVA) was performed at a significance level of 0.05 (*p* < 0.05). Error bars in the figures represent the standard deviation of the measured data.

A strict mixing procedure was followed to ensure homogeneity. The solid materials (cement, sand, and stone/PGA/PG) were dry-mixed in a compulsive mixer for 2 min. Subsequently, water and admixtures were added, and the mixture was stirred for an additional 3 min. The fresh concrete was cast into molds in two layers, with each layer compacted using a vibrating table for 60 s to eliminate entrapped air and prevent segregation. After casting, the molds were covered with plastic film to prevent moisture loss. After curing at room temperature for 24 h, the samples were demolded and placed in a standard curing chamber (temperature: 20 ± 2 °C; relative humidity: ≥95%) for 3, 7, or 28 days. The mechanical properties and microstructures of the samples were subsequently measured.

### 2.3. Test Methods

#### 2.3.1. Microtest

**Figure 5:**
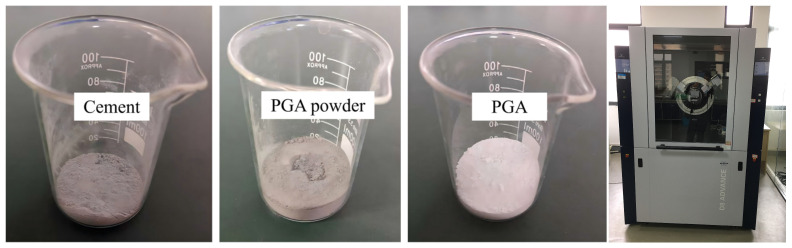
XRD samples and the diffractometer.

The XRD patterns were obtained via a BRUKER D8 ADVANCE XRD diffractometer (Bruker, Karlsruhe, Germany). Prior to testing, the samples were ground into powder and dried, as shown in Figure 5. The diffraction measurement angle range was set from 10° to 70°.

**Figure 6:**
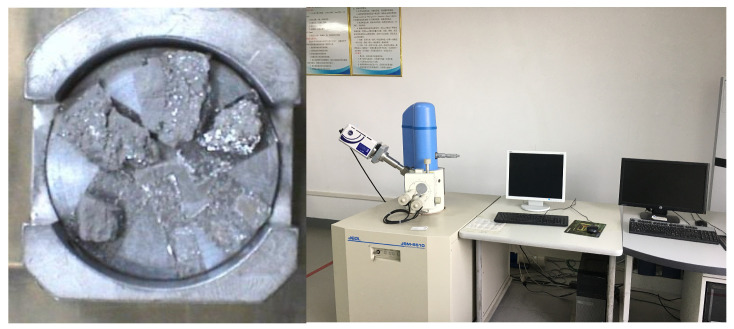
SEM scanning samples and scanner.

The surface microstructure of the concrete samples was analyzed via a JSM-6510 scanning electron microscope (SEM). This instrument was manufactured by JEOL Ltd., Tokyo, Japan, and the samples were taken from the interior of the concrete. To ensure the clarity of the scanning images, the sample surfaces were coated with gold to increase their conductivity, as shown in Figure 6. On the basis of these observations, magnification levels ranging from 2000 to 8000 were selected for scanning.

#### 2.3.2. Mechanical Properties

The flowability of the concrete samples was determined according to the Chinese standard GB/T 50080-2016 [26]. Since the flowability of the prepared samples in this experiment was relatively good, the inverted slump cone emptying test was used to measure the flowability of the concrete mixture. During the test, the concrete was poured into an inverted slump cone, sealed, and then inverted. The time taken for the concrete to completely flow out of the cone was recorded. The shorter the time is, the better the flowability of the concrete. The test was completed within 150 s, and the results were averaged from two tests, which were accurate to 0.1 s.

The water absorption of the concrete samples was determined according to the Chinese standard GB/T 50081-2019 [27]. First, the samples were immersed in water at 20 ± 2 °C for 48 h until they reached a constant mass, ensuring full saturation. The saturated surface-dry mass (*m_s_*) was recorded after wiping the surface moisture with a damp cloth. Subsequently, the samples were placed in a forced-air drying oven at 105 ± 5 °C and dried for approximately 24 h until the mass change between successive weighings at 4 h intervals was less than 0.1%, yielding the oven-dried mass (*m_d_*). The water absorption (*W_a_*) was calculated via the following formula:

(1) $$W_{a}=(m_{s}-m_{d})/m_{d}\times 100\%$$

The compressive strength of the concrete samples was determined according to the Chinese standard GB/T 50081-2019. An electrohydraulic servo pressure tester (capacity: 2000 kN) was used. The specimen was placed centrally on the lower bearing plate of the tester, ensuring that the loading surface was parallel to the curing surface. A preloading step was applied at a rate of 0.2 MPa/s to a load of 10% of the estimated ultimate load, and the load was maintained for 30 s to eliminate gaps between the specimen and the bearing plates. After preloading, the formal loading was conducted at a constant rate of 0.5 MPa/s until the specimen failed. The peak stress was recorded automatically by the tester. Each sample was tested three times. The arithmetic mean of three test results was reported as the compressive strength of the group, with individual values allowed to deviate by no more than ±15% from the middle value; otherwise, the test was repeated. Since the samples in this experiment were of nonstandard dimensions, the obtained strength values were multiplied by a size conversion factor of 0.95.

The stress–strain curves of the concrete samples were tested via the RMT-301 Rock and Concrete Mechanical Testing System (sourced from Institute of Rock and Soil Mechanics, Chinese Academy of Sciences, Wuhan, China) under uniaxial compression, with a device capacity of 1500 kN. During the test, the loading was applied at a constant displacement rate of 0.02 mm/min. Two linear variable differential transformers (LVDTs) were installed on opposite sides of the specimen to record axial deformation, eliminating the effect of platen rotation. The load and strain data were recorded simultaneously to plot the stress–strain curve.

The softening coefficient was determined by comparing the compressive strength of saturated samples to that of dried samples. Two sets of three specimens each (cured for 28 days) were prepared. The first set was immersed in water at 20 ± 2 °C for 48 h to reach a water-saturated state. The second set was oven-dried at 105 ± 5 °C for 48 h to reach a completely dry state. The compressive strength of each set was then tested under its respective condition. The strength of the saturated set was recorded as *f_sat_*, while that of the dry set was recorded as *f_dry_.* The softening coefficient (*k*) of the concrete was subsequently calculated via the following formula:

(2) $$k=f_{sat}/f_{dry}\times 100\%$$

## 3. Results and Discussion

### 3.1. Workability and Setting Characteristics

**Figure 7:**
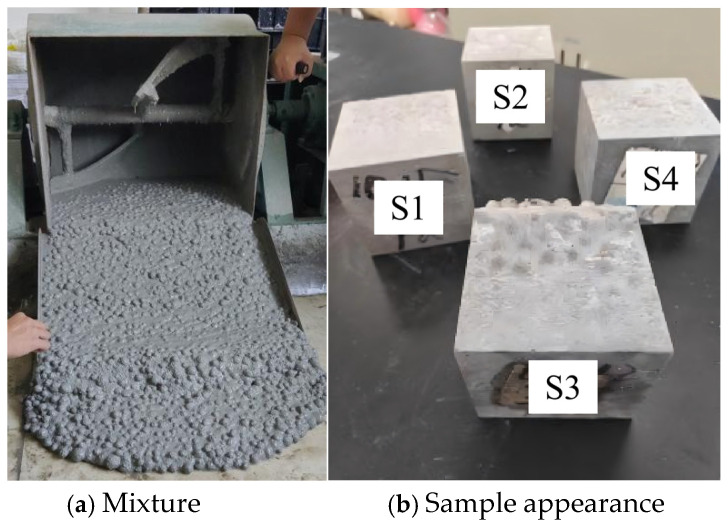
Test samples.

Figure 7a shows the state of the test samples. The excellent fluidity is primarily attributed to the inclusion of a high-efficiency superplasticizer and the spherical morphology of the PGA, which reduces inter-particle friction. The slump was measured via the inverted slump cone emptying test, and the emptying times for the concrete mixtures of S1 to S4 were 15 s, 16 s, 18 s, and 20 s, respectively. The results indicate that the fluidity of S1 to S4 decreases sequentially. Through comparative analysis, it is evident that incorporating an appropriate amount of PG and PGA can enhance the fluidity of the concrete.

Figure 7b shows the state of the concrete after molding. The sample has a grayish-white color with a relatively smooth surface. Both the S1 and S3 samples incorporate the PGA, but compared with S1, S3 is more prone to stratification of the PGA. This stratification in S3 is mainly caused by the significant density differential between the lightweight PGA and the denser cement matrix, leading to aggregate floating during vibration. Stratification not only affects the appearance of the concrete but also tends to cause defects in its mechanical properties. In contrast, the S1 sample includes PG in the cementitious binder, which has a density similar to that of PGA, resulting in minimal stratification.

In addition to workability, the setting behavior and curing requirements were closely monitored given the high dosage of phosphogypsum. It was observed that the mixtures containing high volumes of PG (S1 and S2) exhibited a delayed setting phenomenon compared to the reference cement concrete (S4). The initial and final setting times for the PG-based mixtures were delayed by approximately 6 to 12 h. This retardation is primarily attributed to the presence of soluble impurities in the uncalcined PG, which slow down the hydration process. However, this degree of retardation is considered acceptable for practical engineering applications, as it provides an extended window for transportation and pouring without inducing a flash set. Furthermore, despite the delayed setting, the subsequent strength development was not compromised. All specimens hardened normally under standard curing conditions (20 ± 2 °C, RH ≥ 95%) without the need for modified curing regimes, such as steam curing or heat treatment, proving the cost-effectiveness and feasibility of this material on construction sites.

### 3.2. Failure Mechanism Analysis

**Figure 8:**
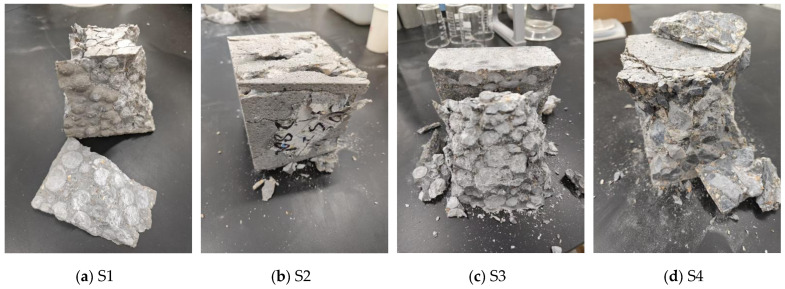
Typical failure modes for each sample.

Analyzing the failure mechanisms of concrete samples is crucial for understanding the mechanical performance of PG concrete under loading. Figure 8 shows typical failure images for each concrete sample under vertical pressure. Upon examining the failed samples, it was observed that the failure modes varied among the different groups.

For S1 (which used both PG and PGA), the primary failure mode was debonding between the aggregate and the paste. Additionally, significant breakage of large-sized PGAs was observed. The top and bottom surfaces of the sample exhibited obvious cracks, and the sides of the concrete experienced extensive spalling and fragmentation under pressure. This finding indicates that the failure of S1 primarily involved interfacial failure between the paste and aggregate, breakage of PGA, and mortar fragmentation.

In contrast, S2 (which replaced PGA with stone) maintained better integrity during failure. The stone aggregate remained unbroken, whereas the concrete surface in contact with the pressure plate experienced spalling. Mortar fragmentation occurred in areas without aggregates, indicating that the failure of S2 mainly involved interfacial failure between the paste and aggregate and mortar fragmentation.

For S3 (which replaced PG with cement but included PGA), the failure surface exhibited obvious stratification. PGA detached from the cement matrix and broke. The cracks on the failure surface were irregular and varied in width, indicating that the failure primarily involved interfacial failure between the paste and aggregate, breakage of the PGA, and minor paste failure.

For S4 (which uses traditional raw materials without PG), the failure involved separation between the aggregate and the paste. The top and sides of the sample cracked and spalled, but the stone aggregate remained relatively intact, with no observed breakage. This finding indicates that the failure of S4 mainly involved interfacial failure between the paste and the aggregate and paste failure.

### 3.3. Density

**Figure 9:**
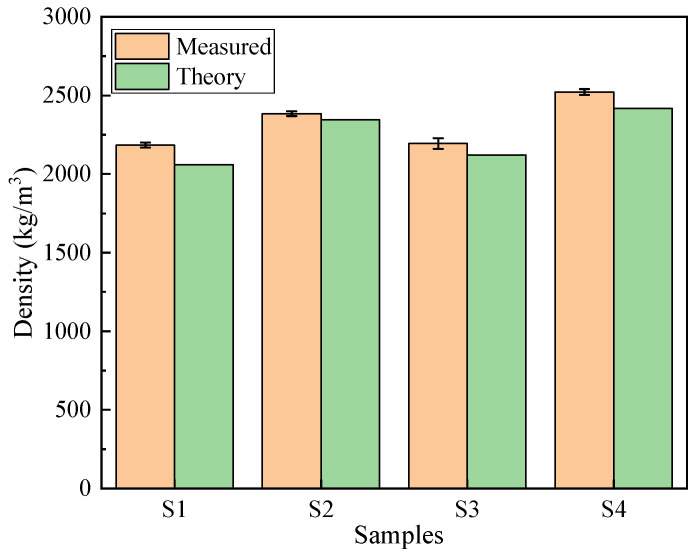
Sample density.

As shown in Figure 9, the densities of the concrete after 28 days of curing are presented. The theoretical density was calculated by summing the masses of all component materials per unit volume as specified in the mix proportions in Table 2. The measured densities for S1 to S4 were 2184.1, 2384.3, 2193.6 and 2521.7 kg/m^3^, respectively. These results indicate that the addition of PG or PGA can effectively reduce the density of concrete. Compared with S4, which uses traditional raw materials, the density of S1 decreased by 13.4%. The maximum relative error between the measured densities and the theoretical densities for S1 to S4 is 6.1%, demonstrating that the material mix design has high reliability compared with the theoretical values. Overall, the incorporation of PG and related products is highly important for the development of lightweight concrete.

### 3.4. Water Absorption

The durability of concrete is closely related to its water absorption characteristics. Generally, the lower the water absorption of concrete is, the better its durability. The water absorption of concrete is primarily determined by its pore structure, and the incorporation of PG can influence this pore structure. The water absorption of the samples was measured via the dry–wet method. The mass difference of the concrete samples in dry and wet states was measured, and the water absorption of the concrete was calculated.

**Figure 10:**
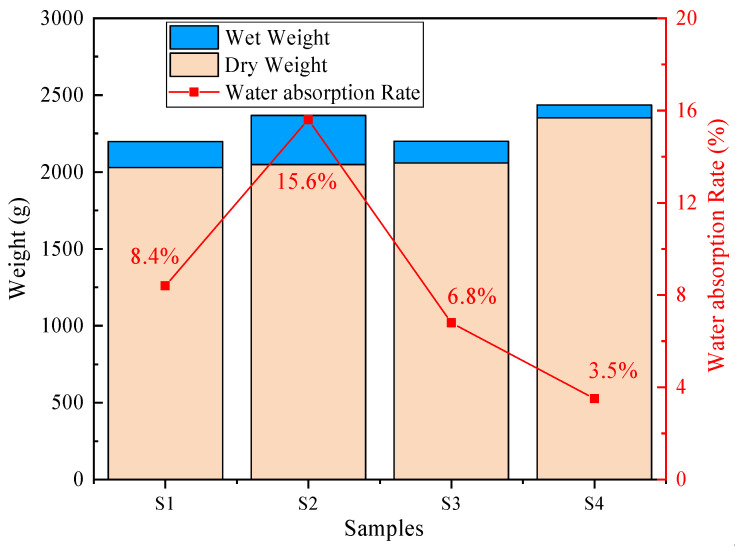
Water absorption rates.

**Figure 11:**
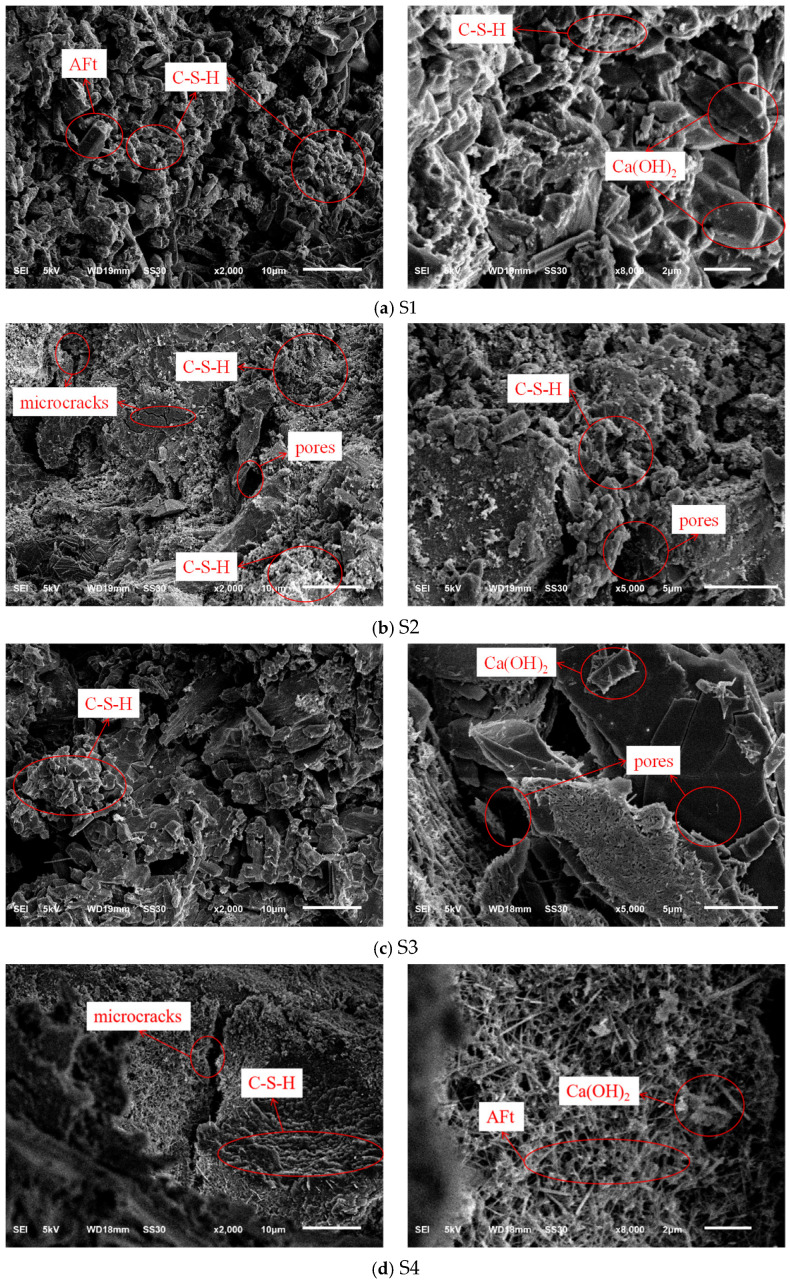
SEM image of the concrete.

As shown in Figure 10, the water absorption test results of the concrete samples indicate that the water absorption rates of the samples follow the order S2 > S1 > S3 > S4, with values of 15.6%, 8.4%, 6.8%, and 3.5%, respectively. The lower water absorption of S3 and S4 is attributed mainly to the higher cement content, indicating that cement as a cementitious binder can form a denser structure. The incorporation of PG in the cementitious binder slightly reduces the compactness of the concrete. Compared with S1 and S2, S1 uses a regular spherical PGA, whereas S2 uses irregular stone aggregates. Although natural stone aggregates inherently possess higher density and lower porosity than PGA, the S1 system surprisingly exhibited lower water absorption than S2. This phenomenon is attributed to the interfacial compatibility rather than the intrinsic density of the aggregates. The chemical homology between the PG binder and PGA in S1 facilitates a continuous and tight bond, thereby enhancing the overall integral compactness of the concrete matrix. Consequently, despite the high density of the stone aggregate itself, the formation of interfacial microcracks and voids creates interconnected pathways that significantly increase water absorption. Although Mercury Intrusion Porosimetry (MIP) would be required to quantify the exact pore size distribution, the significant microcracks and voids observed at the S2 ITZ in the SEM analysis (Figure 11b) provide macroscopic evidence of this connected porous network, which facilitates water ingress.

### 3.5. Microstructure Analysis

Figure 11 presents the microstructural morphology of the concrete samples after 28 days of curing. The SEM micrographs reveal distinct hydration characteristics and interfacial transition zone (ITZ) qualities resulting from the different binder–aggregate combinations. Common hydration products, including flocculent calcium silicate hydrate (C-S-H) gel, hexagonal plate-like calcium hydroxide (Ca(OH)_2_), and needle-like ettringite (AFt), are observed across the samples, but their distribution and interaction with the aggregates vary significantly.

In the reference sample S1 (Figure 11a), where PG is utilized as both binder and aggregate, the microstructure exhibits a high degree of chemical homogeneity. A dense network of needle-like ettringite crystals and C-S-H gel is observed. Notably, the interface between the PG binder matrix and the PGA is indistinct and intimately bonded, suggesting a continuous microstructural development. Since both the binder and the aggregate are sulfate-based materials, they share high chemical compatibility. The PGA surface provides abundant nucleation sites for the hydration products, allowing ettringite crystals to grow into the micro-pores of the aggregate. Based on the SEM morphology, this appears to create a physical-chemical interlocking effect, effectively connecting the matrix and the aggregate. This coherent interfacial bonding helps compensate for the intrinsic porosity of the PGA, ensuring structural integrity. The formation of this dense microstructure is also intrinsically linked to the chemical admixtures used. The superplasticizer ensured a homogeneous dispersion of the binder particles prior to hardening, minimizing the formation of agglomerates and large voids within the matrix. Furthermore, the retarder modulated the hydration rate, preventing the chaotic, rapid precipitation of hydration products. This controlled environment allowed ettringite crystals and C-S-H gels to grow in an organized manner, promoting the development of the interlocked network observed in the ITZ, which is essential for the mechanical performance of the dual-functional system.

In sharp contrast, S2 (Figure 11b), which combines the PG binder with natural stone aggregates, displays visible interfacial defects. Although the stone aggregate itself is dense, significant microcracks and voids are observed at the ITZ. This can be attributed to the chemical incompatibility between the sulfate-rich PG binder and the inert silicate surface of the stone. The rapid formation of expansive ettringite in the PG binder may generate crystallization pressure that the smooth stone surface cannot accommodate, leading to debonding. This weak physical bond explains why S2 failed to achieve higher compressive strength than S1, despite using stronger natural aggregates.

For the cement-based control groups, S3 (Figure 11c) shows a relatively loose microstructure with layered Ca(OH)_2_ and fewer hydration products compared to the pure cement system, correlating with its lower strength. S4 (Figure 11d) exhibits the densest microstructure, characterized by a compact C-S-H gel network interwoven with Ca(OH)_2_ and massive ettringite clusters. Despite the presence of some microcracks, the strong chemical affinity between Portland cement and natural stone results in a classic, robust ITZ, contributing to S4 having the highest absolute strength among all groups.

In conclusion, the microstructural analysis supports the interfacial compatibility hypothesis. While pure cement (S4) provides the highest density, the dual-functional PG system (S1) achieves a unique synergistic effect. The homologous nature of the PG binder and PG aggregate facilitates the formation of a continuous, interlocked microstructure, effectively densifying the ITZ and validating the feasibility of using PG-derived solids in structural concrete applications.

Despite the enhanced mechanical performance driven by the formation of ettringite, the potential risk of sulfate expansion over an extended service life requires careful consideration. The dual-functional system introduces a high concentration of internal sulfate ions. In conventional concrete, excess sulfates often lead to deleterious expansion via delayed ettringite formation (DEF) or secondary gypsum crystallization. However, in this homologous PG-binder/PGA system, the expansive pressure generated during early hydration is effectively utilized to fill the micropores of the aggregate and densify the ITZ, as shown in Figure 11a. The resulting low-permeability structure limits the moisture migration necessary for long-term ionic transport and secondary reaction. Consequently, the volume stability relies on maintaining this dense microstructure to prevent the crystallization pressure from exceeding the tensile strength of the matrix over time. While the current 28-day observation shows a stable, integrated interface, the thermodynamic equilibrium of these sulfate-rich phases under fluctuating environmental conditions warrants long-term monitoring.

It should be noted that while SEM analysis provides qualitative evidence of a densified microstructure and improved ITZ, quantitative porosimetry measurements (such as MIP) were not conducted in this study. However, the macro-performance indicators, specifically the significantly reduced water absorption and higher softening coefficient of the S1 system when compared to the S2 system that suffers from interfacial incompatibility, align with the visual observations of improved interfacial compatibility.

### 3.6. Compressive Strength

**Figure 12:**
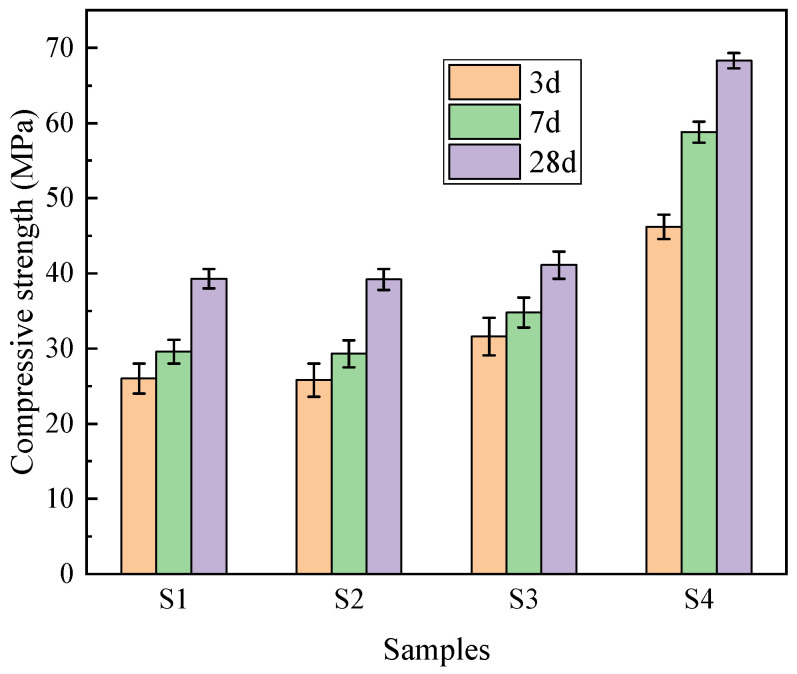
Compressive strength development of concrete samples at 3, 7, and 28 Days.

Figure 12 illustrates the compressive strength development of the concrete samples at 3, 7, and 28 days. The results exhibit a clear differentiation based on the binder–aggregate combinations. The reference group S4 (pure cement and stone aggregate) demonstrated the highest mechanical performance, with a 28-day compressive strength of 68.3 MPa. In comparison, the dual-functional PG utilized sample S1 achieved 3-day, 7-day, and 28-day strengths of 26.0 MPa, 29.6 MPa, and 39.3 MPa, respectively. Although the compressive strength of S1 represents a reduction compared to the conventional high-strength concrete (S4), it is critical to evaluate these results within the context of engineering feasibility and material standards. According to the Chinese standard GB 50010-2010 [28], structural concrete typically requires a strength grade of C30 (30 MPa) or above. The proposed S1 formulation, with a 28-day strength of 39.3 MPa, comfortably exceeds this threshold. This confirms that despite the inclusion of 38% phosphogypsum waste by mass, the material retains sufficient mechanical integrity for general structural applications, validating the feasibility of the proposed dual-functional utilization strategy.

A comparative analysis of S1 and S2 reveals a significant mechanism regarding the compatibility between binder and aggregate. S2 (PG-binder with stone aggregate) and S1 (PG-binder with PGA) exhibited nearly identical 28-day strengths (39.2 MPa vs. 39.3 MPa). Theoretically, natural stone has a much higher intrinsic strength than artificial PGA. The fact that S2 did not outperform S1 indicates that in a PG-based binder system, the aggregate strength is not the limiting factor. Instead, the performance is governed by the strength of the binder matrix and the Interfacial Transition Zone (ITZ).

Comparing S3 (Cement + PGA, 41.1 MPa) and S4 (Cement + Stone, 68.3 MPa) further clarifies the role of the aggregate. When a high-strength cement binder is used, the weaker PGA becomes the limiting factor, leading to a 39.8% reduction in strength compared to S4. However, when a moderate-strength PG binder is used (S1 vs. S2), the aggregate type becomes less significant due to the dominant influence of the binder strength and interfacial bonding.

In summary, while the absolute strength of the PG concrete is lower than that of pure cement–stone concrete, it achieves an effective balance between mechanical compliance and environmental benefits. The results demonstrate that the synergistic use of PG as both binder and aggregate creates a chemically compatible system that mitigates the potential downsides of using artificial waste aggregates.

### 3.7. Stress–Strain Curves

**Figure 13:**
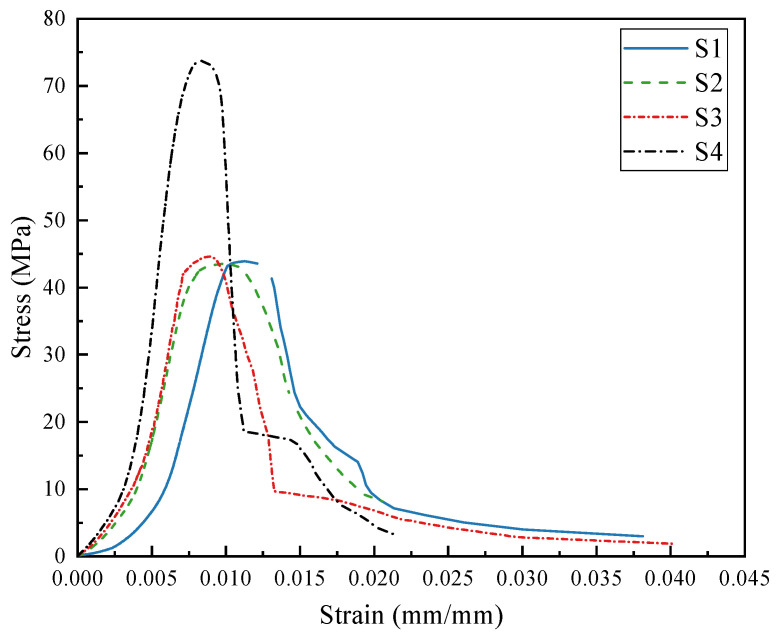
Stress–strain curves of concrete samples at 28 days.

To investigate the influence of PG on the constitutive relationship of concrete, concrete samples cured for 28 days were selected. A gradual increase in load was applied via a compression testing machine until the samples failed, and the pressure and deformation data were recorded. On the basis of these data, stress–strain curves were plotted, as shown in Figure 13. The figure illustrates the stress–strain characteristics of concrete samples S1 to S4. All curves exhibit a development pattern similar to that of ordinary concrete and can be divided into four stages: the elastic stage, the elastoplastic stage, the yield stage, and the failure stage.

In the elastic stage, the stress is directly proportional to the strain, allowing for the calculation of the elastic modulus of the concrete, as presented in Table 3. Specimen S4 has the highest elastic modulus at 18.66 GPa, whereas S1 has the lowest elastic modulus at 8.60 GPa. Compared with that of ordinary concrete, the elastic modulus of S1 is relatively low, primarily due to the lower intrinsic stiffness of the PG-based binder and PGA compared to conventional cement and stone ingredients.

In the elastoplastic stage, the relationship between stress and strain becomes nonlinear. As the load increases, the peak stress of the concrete increases. The peak stresses for S1 to S4 are 43.90 MPa, 43.36 MPa, 44.58 MPa, and 73.69 MPa, respectively, with corresponding peak strains of 11.20%, 9.71%, 8.86%, and 8.35%, respectively. The concrete samples subsequently enter the failure stage, where the stress sharply decreases, the strain significantly increases, and the samples exhibit noticeable cracking and fragmentation.

### 3.8. Softening Coefficient

**Figure 14:**
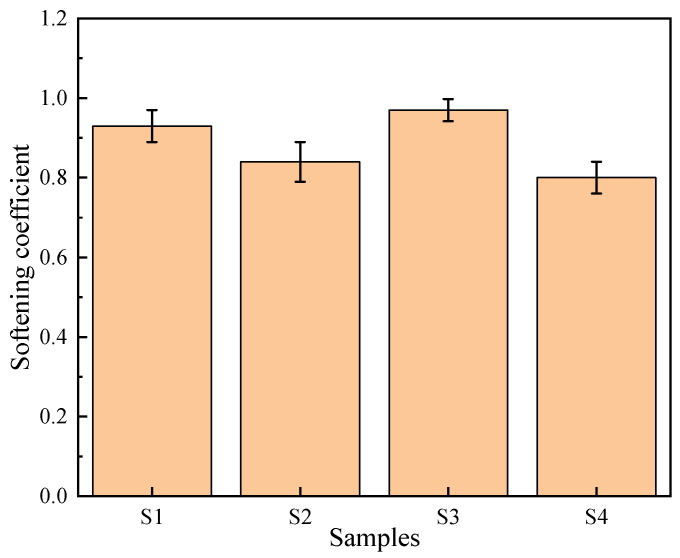
Softening coefficient of concrete samples at 28 days.

The softening coefficient reflects the extent to which the strength of concrete decreases after water absorption and is an important parameter for evaluating the durability of concrete. To investigate the effect of incorporating PG on the softening coefficient of concrete, the softening coefficients of four concrete samples were measured, as shown in Figure 14.

The softening coefficient of S1 is 0.93. Although PG was used as both the cementitious binder and aggregate in S1, its durability performance remained relatively good. The primary reason is that the hydration products, such as ettringite, generated during the hydration of PG fill the pores, increasing the compactness of the structure and thereby improving durability. The softening coefficient of S2 was 0.84, where PG was used as the cementitious binder and stone was used as the aggregate. The durability performance of this combination is moderate, mainly because the interfacial bonding between PG and the stone aggregate is not as tight as that between the PG aggregate and the PG cementitious binder in S1, leading to more pores and microcracks at the interface. The softening coefficient of S3 is the highest, reaching 0.97, indicating that the combination of cement as the cementitious binder and PG as the aggregate performs best in terms of durability. The main reason is that the hydration products of cement form strong bonds with PGA, reducing the porosity and microcracks at the interface, thereby improving its strength retention in wet environments. The softening coefficient of S4 is the lowest, at approximately 0.80. Although the hydration products of cement generally exhibit good bonding properties, the interface between the inert stone and cement paste relies primarily on physical adhesion and weak Van der Waals forces. Unlike the chemically interlocked interface in the PG system, this physical boundary is highly sensitive to moisture. Even minimal water ingress can generate disjoining pressure at the ITZ, significantly weakening the bond strength, thereby resulting in a lower softening coefficient despite the matrix’s overall low water absorption.

While this study primarily evaluated durability through water absorption and softening coefficient tests, these metrics serve as critical indicators for the material’s potential resistance to long-term degradation. The dense ITZ and refined pore structure observed in the dual-functional system (S1), as evidenced by the SEM analysis, imply a reduced permeability that is generally favorable for resisting freeze–thaw cycles and carbonation. However, considering the high sulfate content of phosphogypsum, the risk of secondary ettringite formation and chemical erosion remains a concern for long-term service life. Therefore, establishing a correlation between these short-term physical indicators and long-term durability metrics (such as freeze–thaw resistance and chemical attack) represents a necessary next step to fully validate the engineering reliability of this system.

## 4. Conclusions

This study systematically explores the synergistic mechanism of PG as both a cementitious binder and an aggregate in concrete, revealing the impact of its dual-function application on material properties. Through comparative experiments, the effects of different PG incorporation methods on the microstructure, hydration products, and mechanical properties of concrete were analyzed. The main conclusions are as follows:

(1) The dual-functional utilization strategy effectively achieved a PG incorporation rate of 38%. While this high-volume replacement resulted in lower compressive strength compared to pure cement–stone concrete, it provides a sustainable solution for reducing PG stockpiles.
(2) The PG concrete achieved a 28-day compressive strength of 39.3 MPa and a softening coefficient of 0.93. These results confirm that despite the strength reduction relative to the control, the material retains sufficient mechanical integrity and water resistance to meet standard building codes for structural applications.
(3) Mechanism analysis suggests that the performance of the dual-functional system is governed by interfacial compatibility. The stone aggregate system provides strength through the aggregate’s hardness, whereas the PG system maintains structural integrity through the chemical synergy between the PG binder and PG aggregate, forming a dense ITZ that prevents the premature failure often seen in incompatible composite systems.
(4) SEM analysis supported the proposed interfacial compatibility mechanism. While the stone-PG interface exhibited physical debonding due to chemical mismatch, the homologous PGA-PG system demonstrated a tightly integrated ITZ. The continuous growth of ettringite crystals into the aggregate pores suggests a chemical anchoring effect, which effectively compensates for the lower intrinsic strength of the waste aggregates and ensures the material meets engineering feasibility standards.

This study provides a scientific basis for the application of PG in concrete, demonstrating its significant potential as a sustainable building material. However, limitations remain regarding the comprehensive evaluation of long-term performance in aggressive environments. Specifically, while the water resistance and interfacial compatibility have been validated, long-term durability tests including freeze–thaw resistance, carbonation depth and the monitoring of potential sulfate-induced volume expansion were not covered in the current feasibility analysis. Future research will focus on these aspects to systematically assess the lifecycle stability of dual-functional PG concrete, with specific attention to preventing deleterious expansion in these sulfate-rich systems under varying environmental loads.

## Figures and Tables

**Table 1:** Physical properties of PG.

Properties	Initial Setting Time/min	Final Setting Time/min	2-h Wet Compressive Strength/MPa	Specific Surface Area/(m^2^/kg)	Ture Density/(g/cm^3^)
Results	4	13	6.89	500	2.4

**Table 2:** Mix proportion design.

Samples	Cement/ kg·m^−3^	PG/ kg·m^−3^	Sand/ kg·m^−3^	Stone/ kg·m^−3^	PGA/ kg·m^−3^	Water/ kg·m^−3^	Superplasticizer/ kg·m^−3^	Retarder/ kg·m^−3^
S1	304.2	304.2	810	-	510	206.9	12.16	1.2
S2	304.2	304.2	810	810	-	206.9	12.16	1.2
S3	608.4	-	810	-	510	206.9	12.16	1.2
S4	608.4	-	810	810	-	206.9	12.16	1.2

**Table 3:** Mechanical test results (Mean ± SD).

Samples	Modulus of Elasticity /GPa	Maximum Stress /MPa	Strain Corresponding to Maximum Stress /(mm/mm ×10^−3^)
S1	8.60 ± 0.42	43.90 ± 1.85	11.20 ± 0.48
S2	9.55 ± 0.51	43.36 ± 2.10	9.71 ± 0.55
S3	10.01 ± 0.85	44.58 ± 3.92	8.86 ± 0.87
S4	18.66 ± 0.68	73.69 ± 2.24	8.35 ± 0.58

## Data Availability

The original contributions presented in this study are included in the article. Further inquiries can be directed to the corresponding author.
